# *PPARGC1A* Is a Moderator of Skeletal Muscle Development Regulated by *miR-193b-3p*

**DOI:** 10.3390/ijms23179575

**Published:** 2022-08-24

**Authors:** Manting Ma, Bolin Cai, Shaofen Kong, Zhen Zhou, Jing Zhang, Xiquan Zhang, Qinghua Nie

**Affiliations:** 1 State Key Laboratory for Conservation and Utilization of Subtropical Agro-Bioresources, Lingnan Guangdong Laboratory of Agriculture, College of Animal Science, South China Agricultural University, Guangzhou 510642, China; 2 Guangdong Provincial Key Lab of Agro-Animal Genomics and Molecular Breeding, and Key Laboratory of Chicken Genetics, Breeding and Reproduction, Ministry of Agriculture, Guangzhou 510642, China

**Keywords:** *PPARGC1A*, *miR-193b-3p*, mitochondrial biogenesis, muscle metabolism, skeletal muscle development

## Abstract

Meat production performance is one of the most important factors in determining the economic value of poultry. Myofiber is the basic unit of skeletal muscle, and its physical and chemical properties determine the meat quality of livestock and poultry to a certain extent. Peroxisome proliferator-activated receptor gamma coactivator 1-alpha (*PPARGC1A*) as a transcriptional coactivator has been found to be widely involved in a series of biological processes. However, *PPARGC1A* is still poorly understood in chickens. In this manuscript, we reported that *PPARGC1A* was highly expressed in slow-twitch myofibers. *PPARGC1A* facilitated mitochondrial biogenesis and regulated skeletal muscle metabolism by mediating the flux of glycolysis and the TCA cycle. Gain- and loss-of-function analyses revealed that *PPARGC1A* promoted intramuscular fatty acid oxidation, drove the transformation of fast-twitch to slow-twitch myofibers, and increased chicken skeletal muscle mass. Mechanistically, the expression level of *PPARGC1A* is regulated by *miR-193b-3p*. Our findings help to understand the genetic regulation of skeletal muscle development and provide a molecular basis for further research on the antagonism of skeletal muscle development and fat deposition in chickens.

## 1. Introduction

In vertebrates, skeletal muscle is made up of myofiber and is the largest tissue in the body. The transformation of myofiber type and changes to the myofiber cross-sectional area are the main modes of skeletal muscle development after birth. Numerous processes have been reported to regulate skeletal muscle development, including genetics, environment, and nutrition, but genetics play the least understood of these critical roles [1].

Peroxisome proliferator-activated receptor gamma coactivator 1-alpha (*PPARGC1A*) is a member of the peroxisome proliferator-activated receptor gamma coactivator 1 family and was originally identified as a transcriptional coactivator whose expression closely correlated with adaptive thermogenesis following exposure to cold temperatures [2]. In animals, *PPARGC1A* is widely distributed in various organs and tissues and can interact with a diverse array of transcription factors to regulate numerous aspects of cell physiology [3].

Previous studies have found that *PPARGC1A* helps to regulate cell processes important for adaptive thermogenesis and energy metabolism, including the related functions of glucose uptake, gluconeogenesis, insulin secretion, and mitochondrial biogenesis [4]. As an important regulator of energy metabolism and mitochondrial biosynthesis [5], *PPARGC1A* can participate in skeletal muscle development and fatty acid oxidation by regulating the number and respiration of mitochondria [6,7,8,9]. In mice, the skeletal muscle-specific overexpression of *PPARGC1A* promoted mitochondrial biogenesis and induced the transformation of fast-twitch fibers to slow-twitch fibers [10]. Using PCR-SSCP and DNA sequencing, a single nucleotide polymorphism located in exon 5 of chicken *PPARGC1A* was found to be associated with skeletal muscle fiber types [11]. However, the exact biological function and regulatory mechanism of *PPARGC1A* in chicken skeletal muscle development is still poorly understood.

MicroRNAs (miRNAs) are endogenous noncoding single-stranded RNA molecules that can degrade or inhibit target mRNAs by perfect or imperfect pairing with the 3′ untranslated region (3′ UTR) of their target mRNA and play a critical role in the regulation of gene expression at post-transcriptional levels [12,13]. Despite the fact that recent studies have found that miRNAs are widely involved in the regulation of muscle developmental processes [14,15,16], little is known about the function of miRNAs for the transformation of myofiber types in chickens.

In livestock and poultry, skeletal muscles are the main resources of animal protein, and the growth and development of skeletal muscle directly influences animal meat quantity and quality [17]. Skeletal muscle is composed of different types of myofibers. Different types of myofibers are different in function, biochemical characteristics, and morphological characteristics [18,19,20]. Under certain conditions, different types of myofibers can be transformed. Recently, it is becoming increasingly clear that the composition of myofiber types has an important relationship with muscle quality [21,22]. In our previous RNA-seq study, we found that *PPARGC1A* was differentially expressed between the pectoralis major (PEM; which is mainly composed of fast-twitch fibers) and the soleus (SOL; which has a higher proportion of slow muscle fibers). Here, using lentivirus-mediated *PPARGC1A* overexpression and interference chicken models, we analyzed the biological functions of *PPARGC1A* in muscle oxidative metabolism, intramuscular fat breakdown, the transformation of fast-twitch fibers to slow-twitch fibers, and muscle hypertrophy. Moreover, *miR-193b-3p*, which is highly expressed in fast-twitch fibers, was found to inhibit the expression of *PPARGC1A* by directly binding to the 3′ UTR of *PPARGC1A*. Our study helps to understand the genetic regulation of skeletal muscle development and provides a molecular basis for further research on the antagonism of skeletal muscle development and fat deposition in chickens.

## 2. Results

### 2.1. Identification of Chicken PPARGC1A

**Figure 1:**
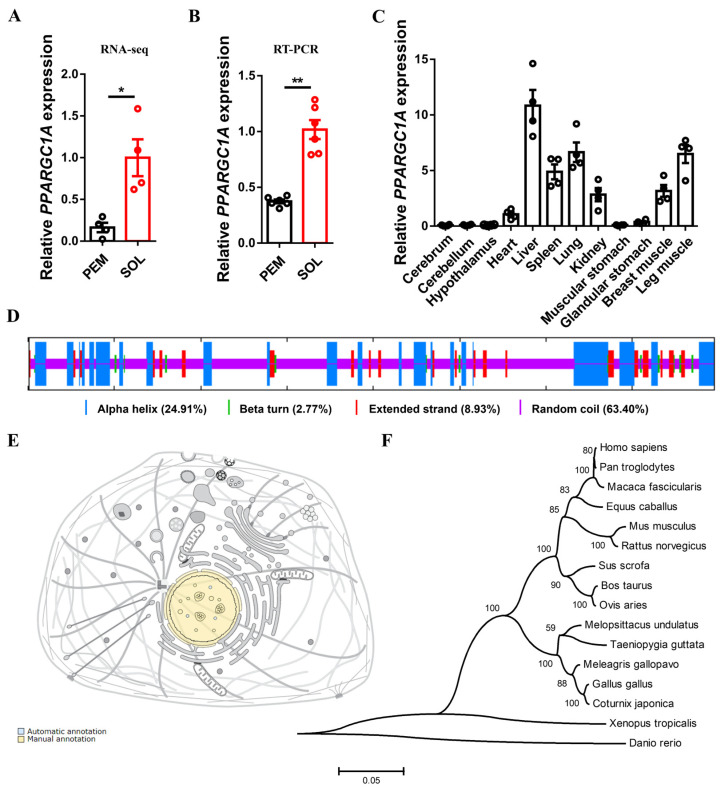
Identification of chicken *PPARGC1A*. (**A**,**B**), relative *PPARGC1A* expression in pectoralis major (PEM) and soleus (SOL) of 7-week-old Xinghua chicken detected by RNA-seq (**A**) and qPCR (**B**). (**C**), tissue expression profiles of *PPARGC1A*. The horizontal axis and vertical axis indicate different tissues and their relative expression values, respectively. (**D**), secondary structure of *PPARGC1A* protein predicted by SOPMA software (https://npsa-prabi.ibcp.fr/cgi-bin/npsa_automat.pl?page=npsa_sopma.html, accessed on 1 February 2022). (**E**), subcellular location of *PPARGC1A* protein annotated by UniProt Knowledgebase (https://www.uniprot.org/, accessed on 1 February 2022). (**F**), phylogenetic tree of chicken *PPARGC1A* aligned amino acid sequences. Results are presented as mean ± SEM. In panels (**A**,**B**), statistical significance of differences between means was assessed using paired *t*-tests. (* *p* < 0.05; ** *p* < 0.01).

Our previous RNA-seq study found that *PPARGC1A* was highly expressed in the SOL (which is an important part of the leg muscles, with a high proportion of slow-twitch fibers) (Figure 1A,B), implying that *PPARGC1A* is probably associated with skeletal muscle development. We examined the tissue expression profile of *PPARGC1A* and also found high expression in liver, lung, breast, and leg muscles (Figure 1C). In order to further analyze the protein structure of *PPARGC1A*, we used SOPMA software to predict its secondary structure. The results showed that the alpha helix, beta turn, extended strand, and random coil accounted for 24.91%, 2.77%, 8.93%, and 63.40% of the *PPARGC1A* protein, respectively (Figure 1D). Furthermore, subcellular location annotation demonstrated that *PPARGC1A* protein exists in the nucleus (Figure 1E), which provides a basis for its interaction with transcription factors. In addition, molecular phylogenetic analysis revealed the chicken *PPARGC1A* protein has a large genetic distance from mammals, whereas it is commonly observed in Aves (such as Coturnix japonica and Meleagris gallopavo) (Figure 1F).

### 2.2. PPARGC1A Facilitates Mitochondrial Biogenesis and Regulates Skeletal Muscle Metabolism

**Figure 2:**
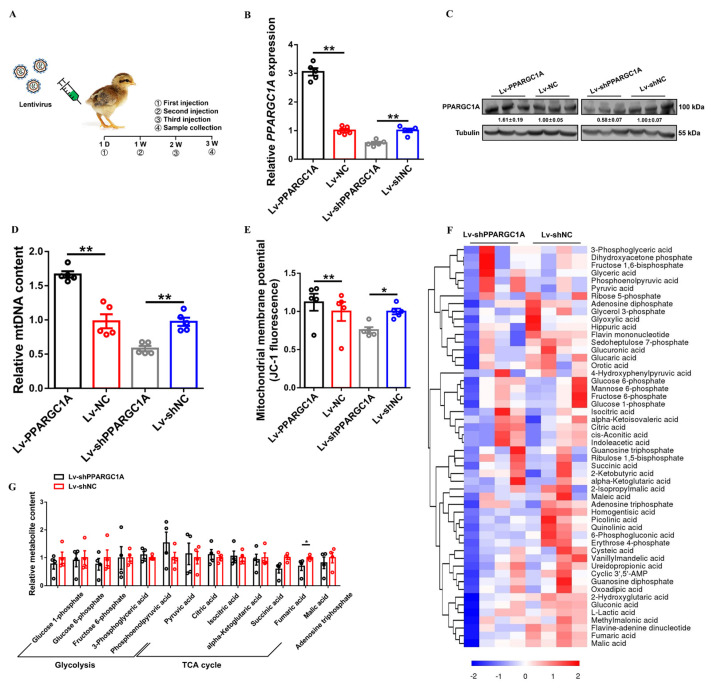
*PPARGC1A* promotes mitochondrial biogenesis and regulates skeletal muscle metabolism. (**A**), construction of lentivirus-mediated *PPARGC1A* overexpression and under–expression chick model. (**B**,**C**), relative *PPARGC1A* mRNA (**B**) and protein (**C**) expression in gastrocnemius muscle with infection of PPARGC1A–expressing lentivirus (Lv–PPARGC1A or Lv–shPPARGC1A) or negative control (Lv–NC or Lv–shNC). (**D**,**E**), relative mitochondrial DNA (mtDNA) content (**D**) and mitochondrial membrane potential (**E**) in *PPARGC1A* overexpression or under–expression gastrocnemius. (**F**), hierarchical clustering analysis (HCA) of gastrocnemius metabolites following Lv–shPPARGC1A or Lv–shNC infection. Relative metabolite levels in *PPARGC1A* under–expression or control group indicated by color. (**G**), relative metabolite content of glycolysis and tricarboxylic acid (TCA) cycle in gastrocnemius following Lv–shPPARGC1A infection. In panels (**C**), ImageJ was used to measure band intensities. The fold change relative to the control is shown below the bands. In panels (**B**,**D**,**E**,**G**), results are shown as mean ± SEM; statistical significance of differences between means was assessed using paired *t*–tests. (* *p* < 0.05; ** *p* < 0.01).

A *PPARGC1A* overexpression vector was constructed and specific small interfering RNAs (siRNAs) against *PPARGC1A* were synthesized to prepare efficient *PPARGC1A* overexpression and under–expression lentiviruses. The qPCR results showed that these vectors and RNA oligonucleotides could significantly overexpress or inhibit the expression of *PPARGC1A* in chicken primary myoblasts (CPMs) (Figure S1A,B). To investigate the biological functions of *PPARGC1A* in the development of chicken skeletal muscle, the gastrocnemius of chicks was injected with a construct mediating lentiviral-mediated *PPARGC1A* overexpression (Lv–PPARGC1A) or lentiviral-mediated *PPARGC1A* under–expression (Lv–shPPARGC1A) at three time points (Figure 2A). Lentiviral-mediated *PPARGC1A* overexpression increased the mRNA and protein levels of *PPARGC1A* in vivo, while *PPARGC1A* mRNA and protein were downregulated after being injected with the *PPARGC1A* under–expression lentivirus (Figure 2B,C).

To investigate the role of *PPARGC1A* in mitochondrial function, we measured the mtDNA content and mitochondrial membrane potential in gastrocnemius injected with the lentivirus. The overexpression of *PPARGC1A* in gastrocnemius increased mitochondrial content as well as mitochondrial membrane potential (Figure 2D,E). By contrast, the mitochondrial content and mitochondrial membrane potential were reduced with *PPARGC1A* under–expression in gastrocnemius (Figure 2D,E).

Given that *PPARGC1A* functions in mitochondrial biogenesis, we performed a comparative metabolome analysis with *PPARGC1A* under–expression in gastrocnemius to further study the regulation of *PPARGC1A* on skeletal muscle metabolism. Hierarchical clustering analysis (HCA) based on metabolite levels showed that *PPARGC1A* under–expression reduced the intermediate products of glycolysis, but facilitated the accumulation of pyruvic acid (which is the end product of glycolysis) (Figure 2F,G; Table S3). Meanwhile, several tricarboxylic acid cycle (TCA cycle) metabolites and adenosine triphosphate (ATP) were actually reduced in *PPARGC1A* under–expression gastrocnemius (Figure 2F,G; Table S3), suggesting that *PPARGC1A* may participate in skeletal muscle development by mediating its metabolism.

### 2.3. PPARGC1A Promotes Intramuscular Fatty Acid Oxidation

**Figure 3:**
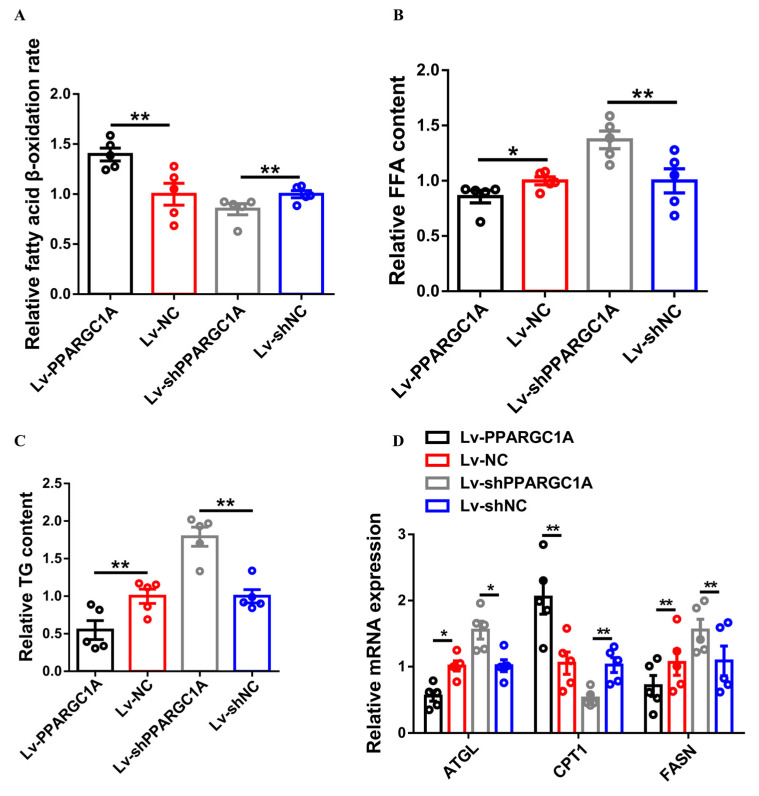
*PPARGC1A* accelerates intramuscular fatty acid oxidation. (**A**) to (**D**), relative fatty acid β–oxidation rate (**A**), relative free fatty acid (FFA) content (**B**), relative triglyceride (TG) content (**C**), and relative mRNA expression levels of fatty acid oxidation or synthesis–related genes (**D**) in gastrocnemius with *PPARGC1A* overexpression or under–expression. Results are presented as mean ± SEM. In all panels, statistical significance of differences between means was assessed using paired *t*–tests. (* *p* < 0.05; ** *p* < 0.01).

We further measured the fatty acid β–oxidation rate to evaluate the role of *PPARGC1A* in intramuscular fat metabolism. *PPARGC1A* overexpression accelerated fatty acid β–oxidation, whereas the fatty acid β–oxidation rate was repressed with *PPARGC1A* under–expression in gastrocnemius (Figure 3A). Furthermore, the FFA and TG content was reduced after the overexpression of *PPARGC1A*, while these metabolites were accumulated in *PPARGC1A* under–expression gastrocnemius (Figure 3B,C). The overexpression of *PPARGC1A* upregulated the expression of FAO-related genes, such as *CPT1*, but downregulated key genes involved in fatty acid synthesis in gastrocnemius, such as *ATGL* and *FASN* (Figure 3D). Moreover, the opposite result was observed in *PPARGC1A* under–expression gastrocnemius (Figure 3D), indicating that *PPARGC1A* can promote intramuscular fatty acid oxidation.

### 2.4. PPARGC1A Activates Slow-Twitch Muscle Phenotype and Induces Muscle Hypertrophy

**Figure 4:**
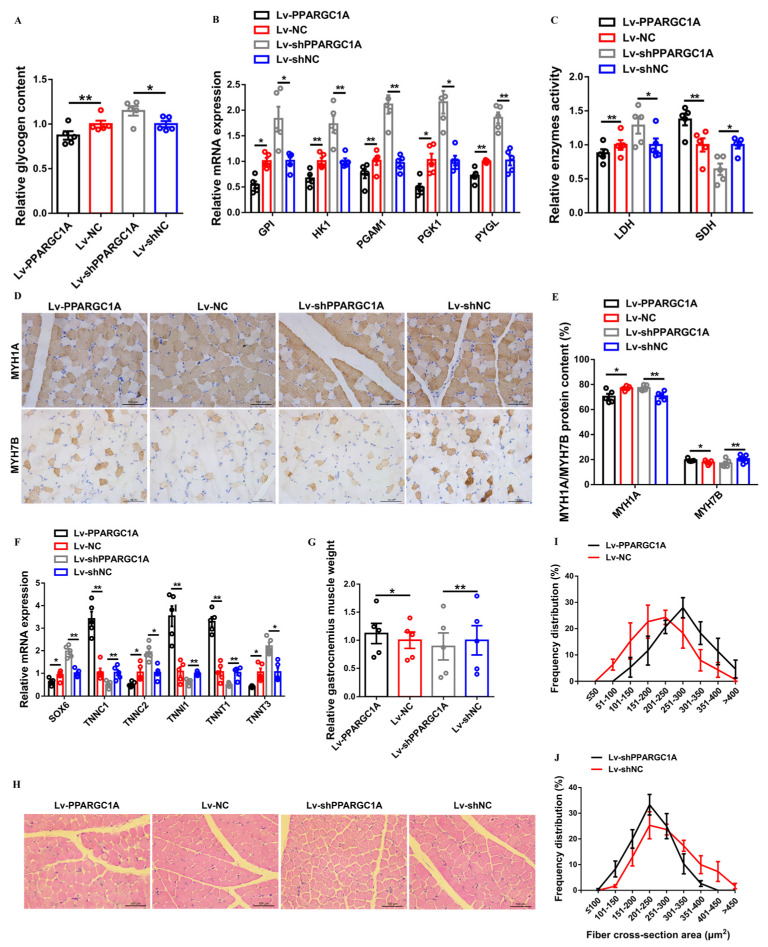
*PPARGC1A* activates slow–twitch muscle phenotype and induces muscle hypertrophy. (**A**–**J**), relative glycogen content (**A**), relative mRNA expression levels of glycogenolytic and glycolytic genes (**B**), relative enzyme activity of lactic dehydrogenase (LDH) and succinate dehydrogenase (SDH) (**C**), immunohistochemistry analysis of MYH1A/MYH7B (**D**), MYH1A/MYH7B protein content (**E**), relative mRNA expression levels of several fast–/slow–twitch myofiber genes (**F**), relative gastrocnemius muscle weight (**G**), H&E staining (**H**), and frequency distribution of fiber cross-sectional area (CSA) (**I**,**J**) in gastrocnemius with *PPARGC1A* overexpression or under–expression. In panels (**A**–**C**,**E**–**G**), results are shown as mean ± SEM, statistical significance of differences between means was assessed using paired *t*–tests. (* *p* < 0.05; ** *p* < 0.01).

Given that *PPARGC1A* overexpression in gastrocnemius increased mitochondrial content and the under–expression of *PPARGC1A* in gastrocnemius repressed the accumulation of TCA cycle metabolites and suppressed oxidative metabolism, we speculated that *PPARGC1A* may modulate muscle metabolism to be involved in the regulation of the transformation of the myofiber type. As expected, *PPARGC1A* overexpression in gastrocnemius decreased glycogen content and repressed the expression of glycogenolytic and glycolytic genes (Figure 4A,B). Conversely, the under–expression of *PPARGC1A* in gastrocnemius facilitated the accumulation of glycogen, as well as upregulating the expression of glycogenolytic and glycolytic genes (Figure 4A,B). The overexpression of *PPARGC1A* suppressed the activity of LDH and enhanced the activity of SDH in gastrocnemius, whereas *PPARGC1A* under–expression in gastrocnemius elevated glycolytic capacity and decreased the oxidative capacity of skeletal muscle (Figure 4C). More importantly, immunohistochemical results showed that the overexpression of *PPARGC1A* in gastrocnemius suppressed the MYH1A/fast–twitch protein level and promoted the expression level of MYH7B/slow–twitch protein (Figure 4D,E). The expressions of multiple slow-twitch myofiber genes, such as *TNNC1*, *TNNI1*, and *TNNT1*, were significantly upregulated, while fast-twitch myofiber genes, such as *SOX6*, *TNNC2*, and *TNNT3*, were suppressed with *PPARGC1A* overexpression in gastrocnemius (Figure 4F). On the contrary, the under–expression of *PPARGC1A* in gastrocnemius upregulated the fast–twitch protein level and the expression of fast-twitch myofiber genes, and this drove the transformation of slow-twitch to fast-twitch myofibers (Figure 4D–F).

Muscle remodeling can also affect muscle mass; this is regulated by anabolic and catabolic signaling pathways, which induce muscle hypertrophy and muscle atrophy, respectively [23]. Here, *PPARGC1A* overexpression increased muscle mass and elevated the proportion of large myofibers (>250 μm^2^) in gastrocnemius (Figure 4G–I). Inversely, gastrocnemius mass was reduced and the proportion of small myofibers (≤300 μm^2^) was increased with *PPARGC1A* under–expression in gastrocnemius (Figure 4G,H,J), suggesting *PPARGC1A* is involved in muscle hypertrophy.

### 2.5. PPARGC1A Is Directly Targeted by miR-193b-3p

**Figure 5:**
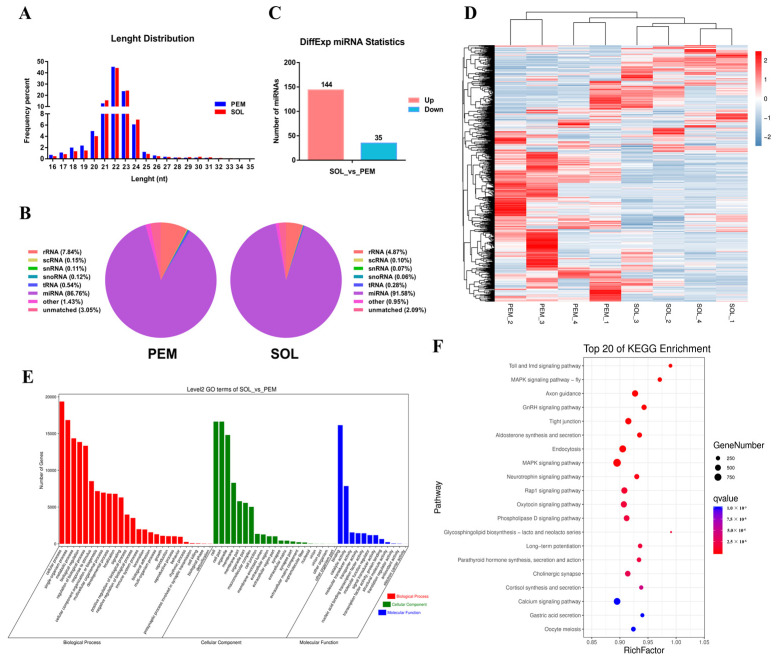
Small RNAs in pectoralis major and soleus of 7-week-old Xinghua chicken. (**A**), length, distribution, and abundance of small RNA sequences in pectoralis major (PEM) and soleus (SOL) of 7-week-old Xinghua chicken. (**B**), distribution frequency of small RNA sequences in different RNA categories. (**C**,**D**), statistics (**C**) and heatmap (**D**) of differentially expressed miRNA between PEM and SOL in 7-week-old Xinghua chicken. (**E**,**F**), GO functions (**E**) and KEGG pathways (**F**) analysis of the target genes of differentially expressed miRNAs.

MiRNAs have been well known to regulate gene expression at post-transcriptional levels. To investigate miRNAs that may target *PPARGC1A*, we analyzed our small RNA sequencing data, which were performed by using the PEM and SOL of a 7-week-old Xinghua chicken. Both in the PEM and SOL, the length of small RNA sequences was mainly concentrated at 21–23 nt, and a length of 22 nt was the maximum size (Figure 5A). Over 85% of the small RNA sequences were identified as miRNAs (Figure 5B). In total, 2156 miRNAs were detected, of which 1313 are known, and 244 are novel. Among them, a total of 179 miRNAs (144 up-regulated in the PEM and 35 in the SOL) were identified as being differentially expressed (DE) between the PEM and the SOL in a 7-week-old Xinghua chicken (|fold change| ≥ 2; *p* value < 0.05) (Figure 5C,D; Table S4). Given that approximately one third of all mammalian genes are thought to be targeted by miRNAs [24], we further predicted the target genes of differentially expressed miRNAs by using RNAhybrid, Miranda, and TargetScan software. A Gene Ontology (GO) analysis found that the target genes of differentially expressed miRNAs were mainly enriched in biological processes such as the cellular process, metabolic process, and biological regulation (Figure 5E). Moreover, a Kyoto Encyclopedia of Genes and Genomes (KEGG) enrichment analysis showed that these target genes participated in the MAPK signaling pathway, RnRH signaling pathway, and calcium signaling pathway (Figure 5F), suggesting that they may play important roles in skeletal muscle development.

**Figure 6:**
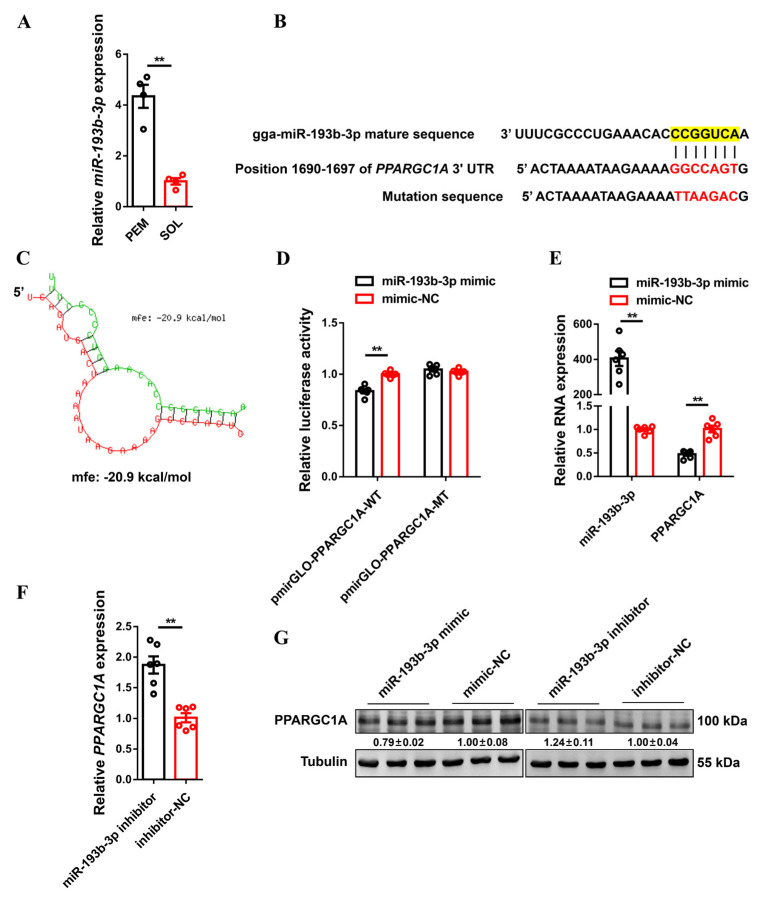
Identification of *PPARGC1A* as a direct target of *miR-193b-3p*. (**A**), relative *miR-193b-3p* expression in pectoralis major (PEM) and soleus (SOL) of 7-week-old Xinghua chicken detected by RNA-seq. (**B**), the potential binding site of *miR-193b-3p* in *PPARGC1A* 3′ untranslated region (UTR). The mutant sequence in *miR-193b-3p* binding site is highlighted in red. (**C**), the potential interaction model between *miR-193b-3p* and *PPARGC1A* from RNAhybrid. (**D**), luciferase assay was conducted by co-transfecting wild-type or mutant *PPARGC1A* 3′ UTR with *miR-193b-3p* mimic or mimic-negative control (NC). (**E**), relative *miR-193b-3p* and *PPARGC1A* expression after *miR-193b-3p* overexpression. (**F**), relative *PPARGC1A* expression after *miR-193b-3p* inhibition. (**G**), the protein expression level of *PPARGC1A* with *miR-193b-3p* overexpression or inhibition. In panel (**G**), ImageJ was used to measure band intensities. The fold change relative to the control is shown below the bands. In panels (**A**,**D**–**F**), results are presented as mean ± SEM, and paired *t*–tests were used to assess the statistical significance of differences between means. (** *p* < 0.01).

*MiR-193b-3p* is highly expressed in fast-twitch myofibers (Figure 6A) and was found to contain potential binding sites for the 3′ UTR of *PPARGC1A* (Figure 6B,C), suggesting that *miR-193b-3p* could be a potential regulatory factor of *PPARGC1A*. To confirm whether *miR-193b-3p* directly targets the 3′ UTR of *PPARGC1A*, a dual-luciferase reporter assay was performed. The results showed that *miR-193b-3p* could perfectly bind with, target, and interact with *PPARGC1A* (Figure 6D). More importantly, *miR-193b-3p* overexpression significantly decreased the mRNA and protein expression level of *PPARGC1A*, whereas the expression of *PPARGC1A* was upregulated with the inhibition of *miR-193b-3p* (Figure 6E,G). In addition, the overexpression of *miR-193b-3p* repressed the expression of FAO-related genes, but upregulated key genes involved in fatty acid synthesis, as well as promoting the expression of glycogenolytic and glycolytic genes and multiple fast-twitch myofiber genes (Figure S2A–C). As expected, the opposite results were observed after *miR-193b-3p* inhibition, which increased *PPARGC1A* expression (Figure S2A–C). Taken together, these results demonstrated that *PPARGC1A* was a direct target of *miR-193b-3p.*

To study whether *miR-193b-3p* plays a similar role in mammalians, we further analyzed the sequence properties of *hsa-*, *mmu-*, and *gga-miR-193b-3p*, and found that they share the same seed sequence (Figure S3A). The interaction between *miR-193b-3p* and *PPARGC1A* in mammalians was also analyzed by the TargetScan software. High binding of *miR-193b-3p* seed sequences to 3′ UTR of *PPARGC1A* was observed (Figure S3B,C), suggesting *miR-193b-3p* is likely to target *PPARGC1A* in mammalians.

## 3. Discussion

As the largest tissue in the body, skeletal muscle is important for broiler production. At the same time, the analysis of the antagonistic effect between muscle development and fat deposition is also a current research hotspot. It is of great significance for poultry production to excavate the genetic regulatory factors involved in skeletal muscle development and clarify their molecular regulatory mechanisms.

Mitochondrial biogenesis is defined as the growth and division of pre-existing mitochondria, thereby increasing the number, size, and mass of mitochondrial population and enhancing mitochondrial function [25]. As the center of the oxidative metabolism, mitochondria have been reported to regulate the metabolic properties of skeletal muscle [26,27]. *PPARδ* is a transcription factor that participates in controlling fatty acid oxidation and energy metabolism [28]. Previous studies have found that *PPARδ* and *PPARGC1A* play important roles in mitochondrial biogenesis [3,29]. *PPARGC1A* could increase the expression of transcription factor A mitochondrial (*TFAM*) to regulate mtDNA transcription and replication by stimulating a series of nuclear transcription factors, such as nuclear respiratory factor 1 (*NRF1*) and nuclear respiratory factor 2 (*NRF2*) [30,31,32]. Moreover, as a transcriptional coactivator, *PPARGC1A* has been found to be widely involved in a series of biological processes by regulating mitochondrial biogenesis and energy metabolism [3,33,34,35]. In this study, we found *PPARGC1A* increased mtDNA content and mitochondrial membrane potential to facilitate mitochondrial biogenesis. Meanwhile, *PPARGC1A* regulated skeletal muscle metabolism by mediating the flux of glycolysis and the TCA cycle.

Excessive intramuscular lipid storage can induce lipotoxic events to affect animal health, as well as cause a decline in meat quality [36,37]. Numerous studies have reported that *PPARGC1A* regulates lipid metabolism and long-chain fatty acid oxidation by upregulating the expression of several genes of the tricarboxylic acid cycle and the mitochondrial fatty acid oxidation pathway [38,39,40]. Here, *PPARGC1A* promoted intramuscular fatty acid oxidation and suppressed fatty acid synthesis, which was potentially attributed to the regulation of *PPARGC1A* in mitochondrial biogenesis and skeletal muscle metabolism.

Myofiber is the basic unit of skeletal muscle; different myofibers have different physicochemical and metabolic characteristics, and their physical and chemical properties determine the meat quality of livestock and poultry to a certain extent [41]. Compared with fast-twitch myofibers, slow-twitch myofibers are rich in hemoglobin and myoglobin, and have higher tenderness, flavor, and juiciness [21,42,43,44]. On the contrary, due to their higher glycogen content and ATPase activity, after slaughter, fast-twitch fibers can cause the pH of muscles to drop rapidly and even produce pale soft exudative (PSE) meat [45,46]. In this study, we found *PPARGC1A* drove the transformation of fast-twitch to slow-twitch myofibers, suggesting that *PPARGC1A* may be a potential target for improving chicken quality.

Skeletal muscle mass is finely regulated by protein synthesis and catabolism [47]. Remarkably, the transformation of myofiber types has been also reported to affect muscle metabolism to regulate muscle mass by changing the metabolic characteristics of myofibers [23]. Here, *PPARGC1A* increased chicken skeletal muscle mass and induced muscle hypertrophy. These results are probably due to changes in skeletal muscle metabolic flux, which lead to an increase in ATP and induce protein synthesis.

It is worth noting that miRNAs have also been demonstrated to play important roles in the development of skeletal muscle by regulating its target genes [14,15,16]. In the current study, we found a total of 179 miRNAs were differentially expressed between the PEM and SOL in a 7-week-old Xinghua chicken, suggesting that these miRNAs may function in the regulation of the transformation of myofibers. Among them, *miR-193b-3p*, which is highly expressed in fast-twitch myofibers, was found to interact with *PPARGC1A*. *MiR-193b-3p* suppressed intramuscular fatty acid oxidation and induced the fast-twitch muscle phenotype, which is partly attributed to its epigenetic regulation of *PPARGC1A*. Interestingly, *hsa-*, *mmu-*, and gga-*miR-193b-3p* share the same seed sequence and potentially the interaction between *miR-193b-3p* and *PPARGC1A* was found in humans and mice, suggesting *miR-193b-3p* is likely to target *PPARGC1A* in mammalians.

## 4. Materials and Methods

### 4.1. Ethics Statement

The Institutional Animal Care and Use Committee at South China Agricultural University approved all animal experimental protocols in this study (approval ID: 2021-C018).

### 4.2. Animals and Cells

One-day-old chicks used in the living experiment were obtained from Kaiping Xufeng Agriculture and Animal Husbandry Co., Ltd. (Kaiping, China).

CPMs were isolated from the leg muscle of 11-day-old chicken embryos as previously described [16]. CPMs were cultured in Roswell Park Memorial Institute (RPMI) 1640 medium (Gibco, Bethesda, MD, USA) with 20% fetal bovine serum (FBS) (Gibco, Bethesda, MD, USA), at 37 °C in a 5% CO_2_ humidified atmosphere.

### 4.3. RNA Extraction, cDNA Synthesis, and Quantitative Real-Time PCR

RNA extraction and cDNA synthesis were performed using Trizol reagent (TaKaRa, Otsu, Japan) and the PrimeScript RT Reagent Kit with gDNA Eraser (Perfect Real Time) (TaKaRa, Otsu, Japan). Quantitative real-time PCR assay was performed as previously described [48]. Primers used for quantitative real-time PCR are listed in Table S1.

### 4.4. Plasmid Construction and RNA Oligonucleotides

For *PPARGC1A* overexpression vector construction, the coding sequence of *PPARGC1A* was amplified and cloned into pcDNA-3.1 (Promega, Madison, WI, USA) between the HindIII and XhoI sites. Specific siRNA against *PPARGC1A* and non-specific siRNA negative control (NC) were designed and synthesized by Guangzhou RiboBio (Guangzhou, China).

For overexpression lentiviral vectors constructed, *PPARGC1A* coding sequence was amplified and then cloned into the pLVX-mCMV-ZsGreen-IRES-Puro vector (Addgene, Cambridge, MA, USA) between the SpeI and NotI sites. Short hairpin RNAs (shRNAs) against *PPARGC1A* were designed and then subcloned into the pLVX-shRNA2-Puro vector (Addgene, Cambridge, MA, USA) between the BamHI and EcoRI sites.

For pmirGLO dual-luciferase miRNA target reporter vectors constructed, the segment sequence of the *PPARGC1A* 3′ UTR that contained the putative *miR-193b-3p* binding sequence was amplified and then cloned into the pmirGLO dual-luciferase reporter vector (Promega, Madison, WI, USA) between the NheI and XhoI sites. Mutant plasmids were generated by changing the binding site of *miR-193b-3p* from GGCCAGT to TTAAGAC.

*MiR-193b-3p* mimic, mimic NC, *miR-193b-3p* inhibitor, and inhibitor NC were designed and synthesized by Guangzhou RiboBio (Guangzhou, China).

The primers and oligonucleotide sequences used in this study are listed in Tables S1 and S2.

### 4.5. Cell Transfection

All transient transfections used Lipofectamine 3000 reagent (Invitrogen, Carlsbad, CA, USA) following the manufacturer’s protocol.

### 4.6. Lentivirus Production and Transduction

Lentivirus production was performed as described before [48]. Thirty 1-day-old chicks were randomly divided into two groups (n = 15): (1) Lv-PPARGC1A and Lv-NC, (2) Lv-shPPARGC1A and Lv-shNC. Chicks received three intramuscular doses (at days 1, 7, and 14) of lentivirus (106 titers) in the lateral head of gastrocnemius muscle. Twenty-one days after the initial injection, chickens were euthanized. Subsequently, gastrocnemius muscles were collected after rapid dissection, then immediately frozen in liquid nitrogen and stored at −80 °C.

### 4.7. Immunoblotting

Western blot analysis was performed as previously described [14]. The primary antibodies used were anti-PPARGC1A (66369-1-Ig; Proteintech, Rosemont, IL, USA; 1:5000), and anti-β-Tubulin (A01030; Abbkine, Waltham, MA, USA; 1:10,000). Goat Anti-Mouse IgG-horseradish peroxidase (HRP) (A21010; Abbkine, Waltham, MA, USA; 10,000) was used as a secondary antibody.

### 4.8. Mitochondrial DNA (mtDNA) Content Assay

A tissue DNA Kit (D3396, Omega, GA, USA) was used to extract total DNA. The amount of mitochondrial DNA was assessed as previously described [16]. Primers used for mtDNA content assay were listed in Table S1.

### 4.9. Mitochondrial Membrane Potential and Fatty Acid Oxidation (FAO) Rate Assay

The mitochondria of gastrocnemius were isolated using the Tissue Mitochondria Isolation Kit (C3606, Beyotime, Beijing, China). After measuring the mitochondrial protein concentration, freshly isolated mitochondria were subjected to mitochondrial membrane potential and FAO rate assay with the mitochondrial membrane potential assay kit with JC-1 (C2006, Beyotime, Beijing, China) and Colorimetric Fatty Acid Oxidation Rate Assay Kit (HL50679, Haling, Shanghai, China), according to the manufacturer’s protocol. The fluorescence and absorbance were determined using a Synergy Neo2 Multi-Mode Microplate Reader (BioTek, Winooski, VT, USA).

### 4.10. Central Carbon Metabolic Profiling

*PPARGC1A* under–expression gastrocnemius samples (n = 4) were used for metabolite extraction, and then performed in HPIC-MS/MS analysis. The high-performance ion exchange liquid chromatography (HPIC) separation was carried out using a Thermo Scientific Dionex ICS-6000 HPIC System (Thermo Fisher Scientific, Waltham, MA, USA). An AB SCIEX 6500 QTRAP+ triple quadrupole mass spectrometer (AB Sciex, Framigham, MA, USA) equipped with an electrospray ionization (ESI) interface was applied for assay development. Metabolic hierarchical clustering analysis (HCA) was performed using Cluster 3.0 software.

### 4.11. Metabolite and Enzyme Activities Assays

Content of free fatty acid (FFA), glycogen and triglyceride (TG), as well as enzyme activity of lactic dehydrogenase (LDH) and succinate dehydrogenase (SDH) in gastrocnemius were measured using commercially available kits (BC0595, BC0345, BC0625, BC0685, and BC0955, Solarbio, Beijing, China) following the manufacturer’s instructions.

### 4.12. Immunohistochemistry and Hematoxylin and Eosin (HE) Staining

An SP-POD kit (SP0041, Solarbio, Beijing, China) was used for immunohistochemistry as recommended by the supplier. The primary antibodies included anti-MYH1A (GTX17485; GeneTex, Irvine, CA, USA; 1:400) and anti-MYH7B (S58; DHSB, Iowa City, IA, USA; 1:100) and were used for labeling the signals.

For HE staining, gastrocnemius muscle tissues were immersed in 4% paraformaldehyde and were then embedded in paraffin and cut into 4 mm-thick transverse sections. Subsequently, the sections were stained using the Hematoxylin and Eosin Staining Kit (C0105S, Beyotime, Beijing, China) according to the manufacturer’s instructions.

### 4.13. Small RNA Sequencing

The PEM and SOL of 7-week-old Xinghua chicken were used for small RNA sequencing. After total RNA was extracted with a TRIzol reagent kit (Invitrogen, Carlsbad, CA, USA), the RNA molecules in a size range of 18–30 nt were enriched by polyacrylamide gel electrophoresis (PAGE). Then, the 3′ adapters were added and the 36–44 nt RNAs were enriched. The 5′ adapters were then ligated to the RNAs as well. The ligation products were reverse transcribed by PCR amplification and the 140–160 bp size PCR products were enriched to generate a cDNA library and sequenced using Illumina HiSeqTM 2500 by Gene Denovo Biotechnology Co. (Guangzhou, China). The raw data of small RNA sequencing were released to the National Center for Biotechnology Information (NCBI) Sequence Read Archive (SRA) database under accession number PRJNA751251.

### 4.14. Dual-Luciferase Reporter Assay

Dual-luciferase reporter assay was performed using a Dual-GLO Luciferase Assay System Kit (Promega, Madison, WI, USA) as previously described [14]. The luminescent signal was quantified using a fluorescence/multi-detection microplate reader (BioTek, Winooski, VT, USA), and firefly luciferase activities were normalized to Renilla luminescence in each well.

### 4.15. Statistical Analysis

Each experiment was repeated at least three times, and all data are expressed as means ± SEM. Where applicable, the statistical significance of the data was tested using independent or paired *t*-tests. The types of tests and the *p* values, when applicable, are indicated in the figure legends.

## 5. Conclusions

In conclusion, we demonstrated that *PPARGC1A*, which is regulated by *miR-193b-3p*, mediates skeletal muscle metabolism to impede intramuscular fat deposition, as well as activating the slow-twitch muscle phenotype and inducing muscle hypertrophy. Our findings help to understand the genetic regulation of skeletal muscle development and provide a molecular basis for further research on the antagonism of skeletal muscle development and fat deposition in chickens.

## Data Availability

The data presented in this study are available on request from the corresponding author.

## Supplementary Materials

The following supporting information can be downloaded at: https://www.mdpi.com/article/10.3390/ijms23179575/s1.
