# Supplementary material for: PPARGC1A Is a Moderator of Skeletal Muscle Development Regulated by miR-193b-3p
Source: Int J Mol Sci. 2022 Aug 24;23(17):9575. doi: 10.3390/ijms23179575 (PMC9455960; doi:10.3390/ijms23179575)
Supplement: Supplementary file 1 [file ijms-23-09575-s001.zip › Supplemental information.pdf]

**Table S1**. Information of Primers.

| Primer name | Primer sequences (5' to 3') | Usage |
|---|---|---|
| qPCR-PPARGC1A | F: AAGCGAAGAGCATTTGTC<br>R: TCGTAGGTGGAGTTAGGC | qPCR |
| qPCR-COX2 | F: GTAGATGCCCAAGAAGTT<br>R: GTTTGATTTAGTCGTCCAG | qPCR |
| qPCR-β-globin | F: CAGCCAGGTGGAGGATTT<br>R: GAATAGGAGGACCCTCTGTTAG | qPCR |
| qPCR-ATGL | F: TGTCCAAAGAAGCACGAAA<br>R: GAGGTATCAGCCCACAGTAGA | qPCR |
| qPCR-CPT1 | F: GCTTATTGTAGTTGTGGGTG<br>R: AAAGTTTGCCGTGTTCAG | qPCR |
| qPCR-FASN | F: CGCAGGCATAGCAGGAAA<br>R: CCAAAGAAGGAGGCATCAA | qPCR |
| qPCR-GPI | F: ATTCACTTTGGGAGCAATC<br>R: ACTCCAACTCTGGCTCAAT | qPCR |
| qPCR-HK1 | F: CTGGATCTCGGTGGTTCTTAC<br>R: TTGTCGGCACGGGAAAGA | qPCR |
| qPCR-PGAM1 | F: GCGAGGCTCAGGTGAAGAT<br>R: GTCCTCCGTCAGGTCAGC | qPCR |
| qPCR-PGK1 | F: CCCTGGATAAGGTGGATG<br>R: TTGTCAGGCATGGGAACT | qPCR |

| | | |
|---|---|---|
| qPCR-PYGL | F: ACATTTGCCTACACGAACC<br>R: TGCCTCCCTCCTCTATCA | qPCR |
| qPCR-SOX6 | F: TCAGGTTCAGGGTCACATGCC<br>R: TTGCTGGAGCTGTAAAGGGC | qPCR |
| qPCR-TNNC1 | F: GTTGAGCAGTTGACAGAAGA<br>R: GAACCATCATAACAAGGAAC | qPCR |
| qPCR-TNNC2 | F: GAGCAGCAAAGATGGCGTCA<br>R: ATCACCGTGCCCAACTCCTT | qPCR |
| qPCR-TNNI1 | F: GAGGAGTGGGAGCAGGAGAT<br>R: TTCGTCCACAATCTCAACCT | qPCR |
| qPCR-TNNT1 | F: GAGCCGCACGGAGAAGGAGC<br>R: CCCGAAGTGGGGCATGTTGG | qPCR |
| qPCR-TNNT3 | F: AGAGGGAAGAAGCAAACAGC<br>R: GTCCCACAGTTCCTTAGCCT | qPCR |
| qPCR-ATROGIN1 | F: TCAACGGGTCGGCAAGTCT<br>R: TCCCTCCCATCGCTCAGTC | qPCR |
| qPCR-MURF1 | F: GGACGAGCGGATCAACAT<br>R: GGGAGATGATGGTCTGGATG | qPCR |
| qPCR-LC3B | F: GAGCAAAGAGTTGAAGATG<br>R: GTCCTAGACGGAAGATTG | qPCR |
| qPCR-SQSTM1 | F: AGCGACGAGGAGCTGGATC<br>R: CCTTGTGGATGCCTTTACCC | qPCR |

| | | |
|---|---|---|
| qPCR-ULK1 | F: TCGTTGCCTTGTATGACTT<br>R: TTTATGCGAATGTTGTTGG | qPCR |
| qPCR-β-actin | F: GATATTGCTGCGCTCGTTG<br>R: TTCAGGGTCAGGATACCTCTTT | qPCR |
| pcDNA3.1- PPARGC1A | F: **AAGCTT**ATGGCGTGGGACATGTGC<br>R: **CTCGAG**TTACCTGCGCAGGCTCCG | Vector construction |
| pDC316-mCMV-ZsGreen-PPARGC1A | F: **ACTAGT**ATGGCGTGGGACATGTGCAACCAGGACTCTGT<br>R: **GCGGCCGC**TTACCTGCGCAGGCTCCGCTGTGCCT | Vector construction |
| pLVX-shRNA2-Puro-PPARGC1A | F: **GATCC**GCAACCAAGATAATCCTTTCTTCAAGAGAGAAAGGATTATCTTGGTTGTTTTT**G**<br>R: **AATTC**AAAAACAACCAAGATAATCCTTTCTCTCTTGAAGAAAGGATTATCTTGGTTGC**G** | Vector construction |

Sequences in bold represent the enzyme cutting sites.

**Table S2.** Oligonucleotide sequences in this study.

| Fragment name | Sequences, 5' to 3' |
|---|---|
| si-PPARGC1A | CAACCAAGATAATCCTTTC |
| miR-193b-3p mimic | AACUGGCCCACAAAGUCCCGCUUU |
| miR-193b-3p inhibitor | AAAGCGGGACUUUGUGGGCCAGUU |

**Table S3**. Comparative metabolome analysis of *PPARGC1A* knockdown versus control group gastrocnemius.

Separate Excel file.

**Table S4.** Differential expression analysis of miRNAs between pectoralis major and soleus in 7-week-old Xinghua chicken.

Separate Excel file.

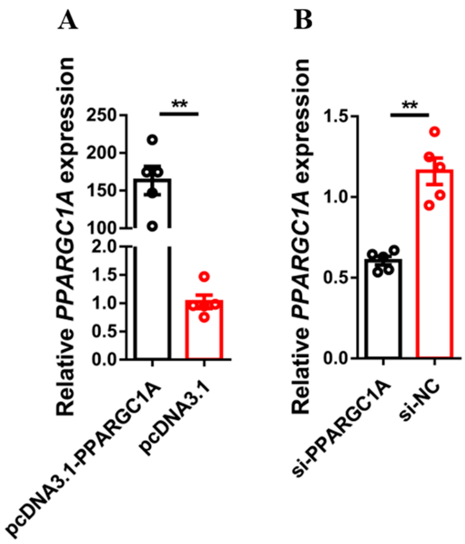


**Figure S1. Analysis of overexpression and interference efficiency of *PPARGC1A in vitro*.** (**A**) Relative *PPARGC1A* expression with *PPARGC1A* overexpression in CPMs. (**B**) Relative *PPARGC1A* expression with *PPARGC1A* interference in CPMs. Results are presented as mean ± SEM. In all panels, statistical significance of differences between means was assessed using independent sample *t*-test. (**$P$ < 0.01).

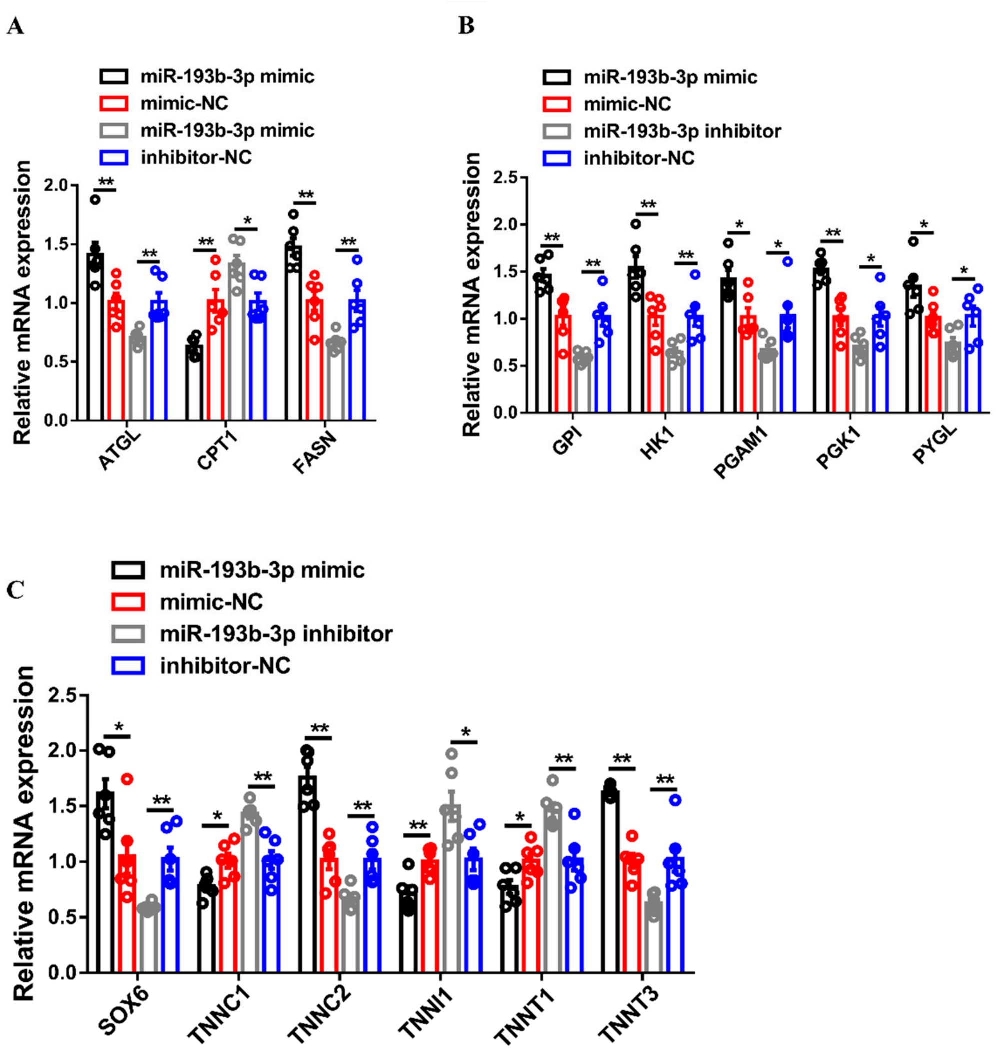


**Figure S2.** ***miR-193b-3p* suppresses intramuscular fatty acid oxidation and induces fast-twitch muscle phenotype.** (**A** to **C**) Relative mRNA expression levels of fatty acid oxidation or synthesis related-genes (**A**), relative mRNA expression levels of glycogenolytic and glycolytic genes (**B**), and relative mRNA expression levels of several fast-/slow-twitch myofiber genes (**C**) after *miR-193b-3p* overexpression or inhibition. In panels (**A** to **C**), results are shown as mean ± SEM, statistical significance of differences between means was assessed using paired *t*-tests. (*$P < 0.05$; **$P < 0.01$).

**A**

gga-miR-193b-3p mature sequence 3' UUUCGCCCUGAAACACCCGGUCAA

hsa-miR-193b-3p mature sequence 3' CGCCCUGAAACUCCCGGUCAA

mmu-miR-193b-3p mature sequence 3' UGACCCUGAAACAUCCGGUCAA

**B** Human

Conserved

| | Predicted consequential pairing of target region (top) and miRNA (bottom) | Site type | Context++ score | Context++ score percentile | Weighted context++ score | Conserved branch length | $P_{CT}$ | Predicted relative $K_D$ |
|---|---|---|---|---|---|---|---|---|
| Position 1754-1760 of PPARGC1A 3' UTR<br>hsa-miR-193b-3p | 5' ...AAGACUAUCUUUGAA-GCCAGUAU...<br>|||||| ||||||<br>3' UCGCCCUGAAACUCCCGGUCAA | 7mer-A1 | -0.21 | 83 | -0.21 | 4.356 | 0.48 | -3.727 |
| Position 1754-1760 of PPARGC1A 3' UTR<br>hsa-miR-193a-3p | 5' ...AAGACUAUCUUUGAA-GCCAGUAU...<br>||||| ||||||<br>3' UGACCCUGAAACAUCCGGUCAA | 7mer-A1 | -0.19 | 82 | -0.19 | 4.356 | 0.48 | -3.775 |

**C** Mouse

Conserved

| | Predicted consequential pairing of target region (top) and miRNA (bottom) | Site type | Context++ score | Context++ score percentile | Weighted context++ score | Conserved branch length | $P_{CT}$ | Predicted relative $K_D$ |
|---|---|---|---|---|---|---|---|---|
| Position 1790-1796 of PPARGC1A 3' UTR<br>mmu-miR-193b-3p | 5' ...AAGACUAUCUUUGAA-GCCAGUAU...<br>||||| ||||||<br>3' UCGCCCUGAAACACCCGGUCAA | 7mer-A1 | -0.19 | 81 | -0.19 | 4.356 | 0.48 | N/A |
| Position 1790-1796 of PPARGC1A 3' UTR<br>mmu-miR-193a-3p | 5' ...AAGACUAUCUUUGAA-GCCAGUAU...<br>||||| ||||||<br>3' UGACCCUGAAACAUCCGGUCAA | 7mer-A1 | -0.19 | 81 | -0.19 | 4.356 | 0.48 | N/A |

**Figure S3. *miR-193b-3p* is likely to target to *PPARGC1A* in mammalian.** (**A**) Sequences of *gga-*, *has-*, and *mmu-miR-193b-3p*. (**B and C**) The interaction between *miR-193b-3p* and *PPARGC1A* in mammalians analyzed by TargetScan.
